# Impact of Vehicular Networks on Emergency Medical Services in Urban Areas

**DOI:** 10.3390/ijerph111111348

**Published:** 2014-10-31

**Authors:** Chun-Liang Lee, Chung-Yuan Huang, Tzu-Chien Hsiao, Chun-Yen Wu, Yaw-Chung Chen, I.-Cheng Wang

**Affiliations:** 1 Department of Computer Science and Information Engineering, School of Electrical and Computer Engineering, College of Engineering, Chang Gung University, No. 259, Wen-Hwa 1st Road, Kwei-Shan, Tao-Yuan 33302, Taiwan; E-Mails: cllee@mail.cgu.edu.tw (C.-L.L.); b0029018@stmail.cgu.edu.tw (I.-C.W.); 2 Department of Computer Science, Institute of Biomedical Engineering, National Chiao Tung University, 1001 University Road, Hsinchu 30010, Taiwan; E-Mails: labview@cs.nctu.edu.tw (T.-C.H.); xxjyen@gmail.com (C.-Y.W.); 3 Department of Computer Science, National Chiao Tung University, 1001 University Road, Hsinchu 30010, Taiwan; E-Mail: ycchen@cs.nctu.edu.tw

**Keywords:** emergency medical services, cumulative survival ratio, vehicular *ad hoc* networks

## Abstract

The speed with which emergency personnel can provide emergency treatment is crucial to reducing death and disability among acute and critically ill patients. Unfortunately, the rapid development of cities and increased numbers of vehicles are preventing emergency vehicles from easily reaching locations where they are needed. A significant number of researchers are experimenting with vehicular networks to address this issue, but in most studies the focus has been on communication technologies and protocols, with few efforts to assess how network applications actually support emergency medical care. Our motivation was to search the literature for suggested methods for assisting emergency vehicles, and to use simulations to evaluate them. Our results and evidence-based studies were cross-referenced to assess each method in terms of cumulative survival ratio (CSR) gains for acute and critically ill patients. Simulation results indicate that traffic light preemption resulted in significant CSR increases of between 32.4% and 90.2%. Route guidance was found to increase CSRs from 14.1% to 57.8%, while path clearing increased CSRs by 15.5% or less. It is our hope that this data will support the efforts of emergency medical technicians, traffic managers, and policy makers.

## 1. Introduction

Heart disease, cerebrovascular disease, and accidental injuries are major causes of death in many countries. Rapid medical treatment can improve prognoses and reduce disabilities among victims. Especially for cardiac arrest patients, receiving timely treatment is key to survival [1,2,3,4]. The functions of emergency medical service (EMS) teams, which are often the first care givers at accident scenes, include: (a) on-site medical treatment for patients with serious or emergency injuries; (b) emergency aid en route to a hospital; and (c) referrals for patients with major injuries living on outlying islands and in remote areas [5]. A large number of studies have shown that the rapid arrival of emergency vehicles (EVs) and the provision of emergency treatment at accident scenes are critical to reducing incidents of death and disability for acute and critically ill patients [6,7,8,9,10,11]. For cardiac arrest patients, survival rates are approximately 43% when basic life support is implemented within 4 minutes, and when advanced cardiac life support is provided within 8 minutes [8]. Patient survival odds are thought to increase by 0.77 for every one minute reduction in arrival time [1].

Rapid development in cities and suburbs are contributing to substantial increases in the numbers of vehicles that can potentially hinder EV movement, a problem made worse by traffic accidents, competing emergencies, and bottlenecks at traffic lights [12]. Intersections with and without traffic control signals and signs also put emergency care providers at risk of becoming accident victims themselves.

A vehicular network (VN) is an emerging technology that supports communication between vehicles via other vehicles or infrastructure [13]. Using a VN, a vehicle can periodically transmit its location, driving speed, or warning messages to other vehicles or a traffic control center to implement intelligent transportation system (ITS) applications. They can be used to provide real-time traffic information for identifying efficient navigation paths. While many researchers have attempted to use VNs to resolve EV-hindrance problems, the majority have focused on communication technologies and protocols, with only a few attempts made to determine the benefits of VNs when applied to emergency medical care [14,15,16,17].

Barrachina *et al.* [15] have proposed four vehicle routing approaches to reducing EV arrival time at accident scenes. Two are based on the Dijkstra algorithm, and two on evolution strategies. While not directly related to emergency services, two other studies have produced results that can be used to assist in achieving EV goals. Fogue *et al.*’s [16] model for sanitary resource allocation in traffic accidents uses multi-objective genetic algorithms. Their goal is to take information gathered from vehicular networks and accident severity estimates, and to apply them so as to achieve optimal resource allocation from limited vehicle and medical staff resources. In [17], the same authors propose an intelligent system involving data mining and knowledge inferences to estimate accident severity. In their paper they present a prototype of the proposed system.

VNs are expensive, therefore implementing them and equipping all vehicles with VN connections is not feasible for many local governments. To increase efficiency and to perhaps reduce the costs of installing VNs, simulations can be used to evaluate their functions and to assess their benefits for emergency medical treatment provision.

Our focus in this paper is to review methods for assisting EVs as found in the literature, especially those methods using VN technology and global positioning systems (GPSs). We performed simulations of various methods to assess CSR gains for acute and critically ill patients. Simulation results also provided data on the extent to which EV travel time can be reduced, and concerning links between road congestion and EV travel time.

The rest of this paper is organized as follows: communications technology and available methods for reducing EV travel time are reviewed in the next section, and the systems implemented in this study and details regarding their operational processes are explained in Section 3. Section 4 focuses on system performance evaluation, including the simulation environment, parameter settings, the simulation process, and a discussion of simulation results. A conclusion is offered in Section 5.

## 2. Related Work

### 2.1. 802.11p

IEEE 802.11p [18], a communications protocol extended from IEEE 802.11, is primarily used in vehicular wireless communications equipment for applications involving intelligent transportation systems (ITS). IEEE 802.11p has three primary characteristics:

1. Interoperability and compatibility with numerous dedicated short-range communication (DSRC) standards, including ITS-dedicated communication standards E2213-02, CALM M5, and IEEE 802.11a. This compatibility gives IEEE 802.11p exceptional market acceptance.
2. High-speed mobility, unlike many DSRC technologies that are incapable of implementing high-speed mobile access.
3. Support from IEEE and the U.S. Department of Transportation, which is responsible for infrastructure construction.

IEEE 1609 is a communications system architecture and series of standardized services and interfaces for radio access applications involving wireless access in vehicular environments (WAVE). Its main application is formulating standard vehicle-to-vehicle (V2V) and vehicle-to-infrastructure (V2I) wireless protocols, primarily for use with car security systems, enhanced navigation, traffic management, automatic fare collection, and similar application scenarios.

**Figure 1:**
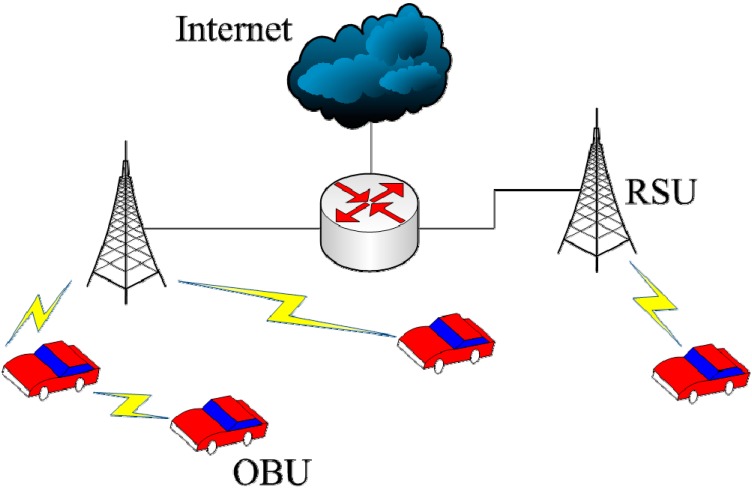
WAVE application architecture.

The IEEE 1609 standard has the IEEE 802.11p communications protocol as its foundation. Current standards formulated under IEEE 1609 include IEEE 1609.0 [19], IEEE 1609.1 [20], IEEE 1609.2 [21], IEEE 1609.3 [22], IEEE 1609.4 [23], IEEE 1609.11 [24], and IEEE 1609.12 [25]. Of these, IEEE 1609.3 defines network and transport layers in the open systems interconnection network model to provide WAVE/DSRC network services. As shown in Figure 1, this enables communications between the onboard units (OBUs) of two vehicles, or between a vehicle’s OBU and a roadside unit (RSU).

### 2.2. Proposed Methods for Reducing EV Travel Time

Numerous methods have been proposed for reducing EV travel time and to enhance first aid efficiency. This paper examined and collected the methods suggested by other recent studies and divided them into three categories: (1) path clearing, (2) route guidance, and (3) traffic light preemption.

#### 2.2.1. Path Clearing

In order to let EVs quickly pass congested areas, EVs can actively disseminate emergency messages to attract the attention of other vehicles along EV routes, thus giving them the opportunity to yield [26,27,28]. According to the method proposed in [26], all vehicles are assumed as having electronic maps and GPS systems. When an EV departs its base station, it uses an electronic map to establish a route, which it follows while broadcasting emergency messages that include EV type, identification code, its planned route, its current position, and average speed. Drivers of vehicles receiving these messages can calculate when they will encounter the EV and make the necessary adjustments to let the EV pass. The primary drawback of this proposal is that the authors only use navigation software to calculate the shortest driving route, and do not consider real-time traffic conditions or traffic light controls.

In a separate paper [27], the authors note that EVs are eight times more likely than other vehicles to be involved in traffic accidents, mostly when other drivers react incorrectly due to confusion over the location of EV flashing lights and sirens. They therefore propose a method for disseminating emergency messages that makes use of OBU monitors to show alerts and to instruct drivers to slow down or move to the side of the road. Emergency messages are disseminated using multi-hop inter-vehicle communication. Finally, in the method described in [28], an EV continuously emits emergency messages to inform a centralized server of its position. The server has all traffic information and is aware of the path that the EV will pass by. Thus, it can send messages at appropriate time to inform RSUs along the travel path of the EV. When RSUs receive the message, they will broadcast warning messages to inform nearby vehicles that an EV will soon pass them.

#### 2.2.2. Route Guidance

In three other papers, EVs are described as using real-time traffic information for navigation to avoid congested road sections. In [28], a centralized server controls all traffic lights and traffic information. Before departing the base station, the EV personnel ask the server to create an enhanced travel route. A shortest-time algorithm is used to plan primary and alternative routes based on distances and average expected speeds. In [29], the authors describe a modified A* algorithm called a learning routing algorithm (LRA) that uses a mix of real-time traffic and EV travel data. They report that LRA execution time is faster than that of the original A* algorithm [30]. The main issue with these studies is their lack of information on (a) how other vehicles should yield to EVs after receiving emergency messages, (b) dealing with traffic lights, and (c) how EVs should respond when they encounter unexpected situations such as other car accidents or traffic congestion along planned routes.

#### 2.2.3. Traffic Light Preemption

In the next set of studies, the authors look at ways to manipulate traffic lights to increase EV route efficiency. In [29], real-time traffic data are collected by telemetry units installed in EVs and traffic lights, with an adaptive A* routing algorithm used to find minimum travel time paths. Real-time traffic data are used to train the neural network and to increase the speed of the routing protocol. At a certain distance, the EV emits a message to change all traffic lights to red. In [31], the authors propose a traffic light preemption algorithm based on cooperative awareness messages (CAM). The authors suggest that spatial and angular information obtained from CAM messages can be used to reduce the probability of errors in traffic preemption. In [32], the authors use state charts to model a traffic light control system with EV preemption, and propose a traffic light preemption policy so that EVs can move with minimum delays.

## 3. System Implementation

### 3.1. System Overview

**Figure 2:**
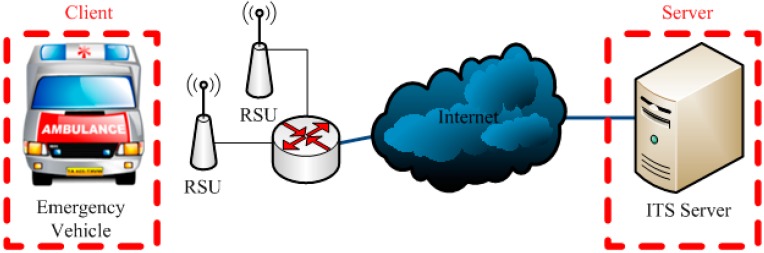
System architecture.

The system architecture used for the present project is shown in Figure 2. The client consists of the EV and WAVE/DSRC communications equipment, and the server is an ITS server. RSUs serve as communication bridges. Their individual functions are as follows:

1. EV equipment consists of a GPS receiver (used to obtain location data) and an OBU (WAVE/DSRC) to send messages to other vehicles and RSUs.
2. The ITS server controls all traffic information and provides real-time traffic data to the EV. The server also alters traffic lights as necessary to enable the EV to quickly move through intersections.
3. RSUs installed along roadsides have two functions: collecting data such as the number of vehicles and their average speeds and feeding them to the ITS server, and supporting communications between the EV and the ITS server. Note that the average speeds of vehicles can be obtained by the ITS server in different ways. For example, each vehicle can transmit its current speed to the ITS server through other vehicles using V2V communication. This paper, however, assumes a RSU can detect the average speed of nearby vehicles and transmits the speed information to the ITS server. This assumption is fairly realistic in urban areas. For instance, a large number of vehicle detectors have been deployed in Taipei City. Currently, these detectors provide real-time traffic information, such as average vehicle speed, for the traffic control center. In the future, it is very likely that RSUs will collocate with existing vehicle detectors for getting access to the Internet and power supply.

**Figure 3:**
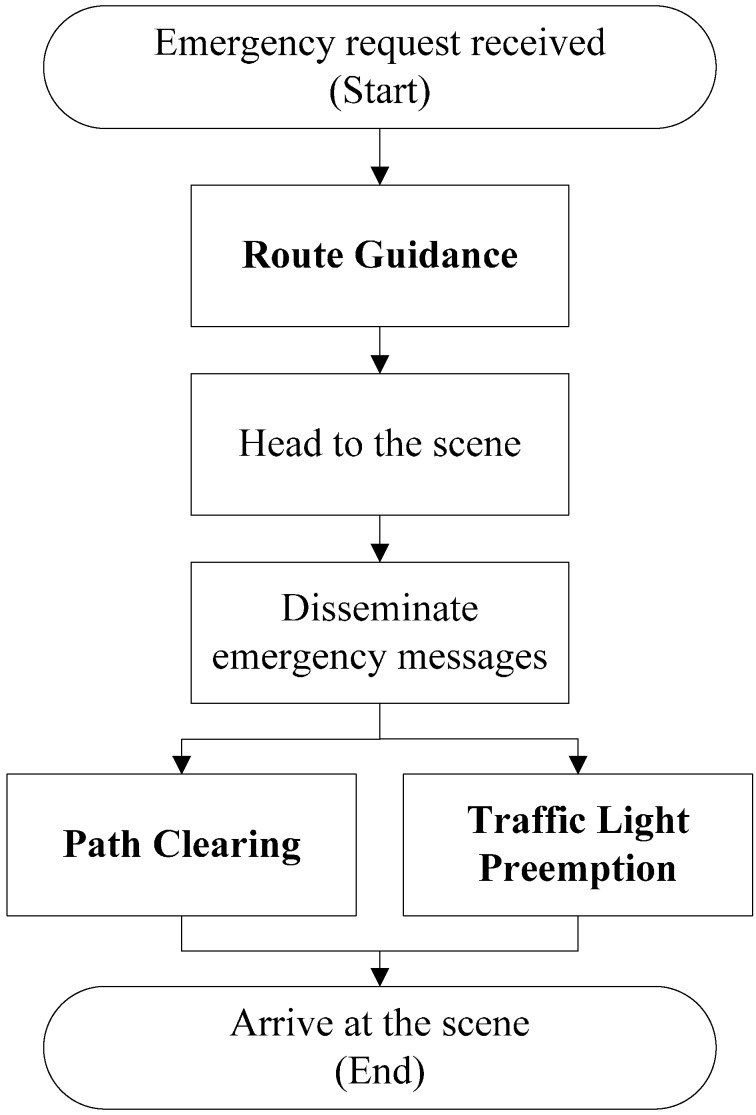
System flow.

The three primary functions of the system proposed in this paper are route guidance, traffic light preemption, and path clearing (Figure 3). When an EV receives an emergency request, it proceeds as follows:

1. If the routing guidance function is enabled, the EV will send a request to the ITS server to ask for a shortest-time path to its destination.
2. The EV periodically broadcasts emergency messages when travelling, until it reaches its destination. An emergency message includes information about the EV, such as its type (e.g., ambulance or fire engine), identification code, planned route, current position, and average speed.
3. If the path clearing function is enabled, vehicles that receive emergency messages will clear a lane for allowing the EV to quickly pass congested areas.
4. If the traffic light preemption function is enabled, the ITS server will control RSUs along the travel route of the EV at appropriate time based on the information sent by the EV.

### 3.2. Route Guidance (RG)

Using route guidance, the EV uses the traffic data collected by the RSUs and the shortest-time algorithm for navigation. This prevents the EV from entering regions with traffic jams. The detailed operational steps are as follows:

1. When an incident occurs, the EV sends information to the ITS server as it departs and asks for route-finding assistance.
2. The ITS server uses the location of EV as the starting point and the site of the incident as the endpoint, and then notifies all RSUs within this range.
3. After receiving a message, the RSUs start monitoring traffic information and sending data to the ITS server, which uses a shortest-time algorithm, such as A* algorithm [30] or Dijkstra algorithm, to find a travel path for the EV.
4. When traveling, the EV regularly broadcasts emergency messages to inform the ITS server of its position. The ITS server then transmits the updated travel path information to the EV.

The shortest-time algorithm used in this paper is A* algorithm. Each intersection is treated as a node of a graph, while each road segment is treated as an edge. The cost of an edge is calculated by dividing the length over the average speed. In order to take the possible delay caused by traffic lights into consideration, the average waiting time at a traffic light, which is set to half of the duration of a red-light period, is added to the cost of each edge. Note that it makes no big difference if A* algorithm is replaced by another shortest-path algorithm. Finding the shortest-time path with real-time traffic information is an interesting and active research topic, but it is beyond the scope of this paper. Here, we focus on how much route guidance contributes to emergency medical services.

### 3.3. Traffic Light Preemption (TLP)

**Figure 4:**
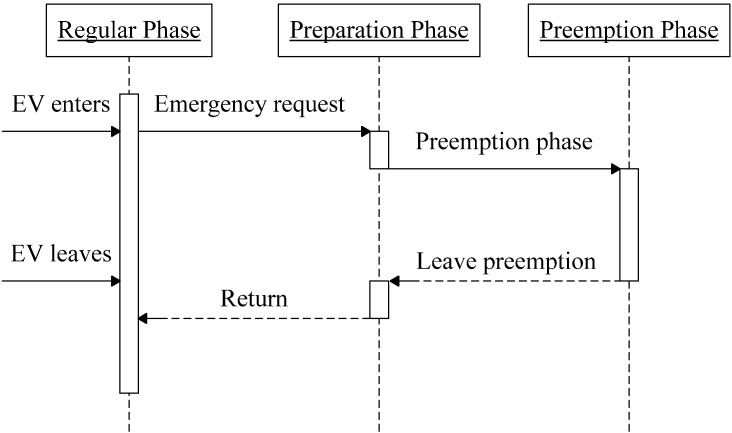
Traffic light preemption timing diagram.

The ITS server can use messages sent by the EV to control traffic lights along its route. Traffic light status can be divided into three phases: regular, with original operational settings in the absence of emergency events; preparation, during which an emergency event is acknowledged; and preemption, in which the EV passes through intersections where traffic lights are all red to stop normal traffic. In the traffic light change process shown in Figure 4, the EV continuously broadcasts emergency messages to the RSUs, which forward them to the ITS server, which in turn uses the data to determine the position of the EV and to control appropriate traffic lights from the preparation to preemption to preparation to regular phases. Since vehicles that previously had a green light are put in a difficult decision-making position as an EV approaches, there is potential for serious accidents to occur. We therefore set the time for the preparation phase to 5 seconds for vehicles to react to unexpected light changes.

### 3.4. Path Clearing (PC)

The PC function gives warnings to other vehicles via emergency messages originating from the EV. The messages contain the following information:

1. The unique identification code of the EV.
2. The vehicle information (*i.e.*, position, direction, and speed) of the EV.
3. The time the emergency message was sent.

Each EV has a unique identification code. This distinguishes various EVs within the same region. Upon receiving the emergency messages, other vehicles can ascertain the EV position on their electronic maps and prepare for evasive action. Compared to the lights and sirens that EVs currently feature, vehicles can better understand the positions and distances between themselves and EVs. The electronic map can convert geographical information into street information.

**Figure 5:**
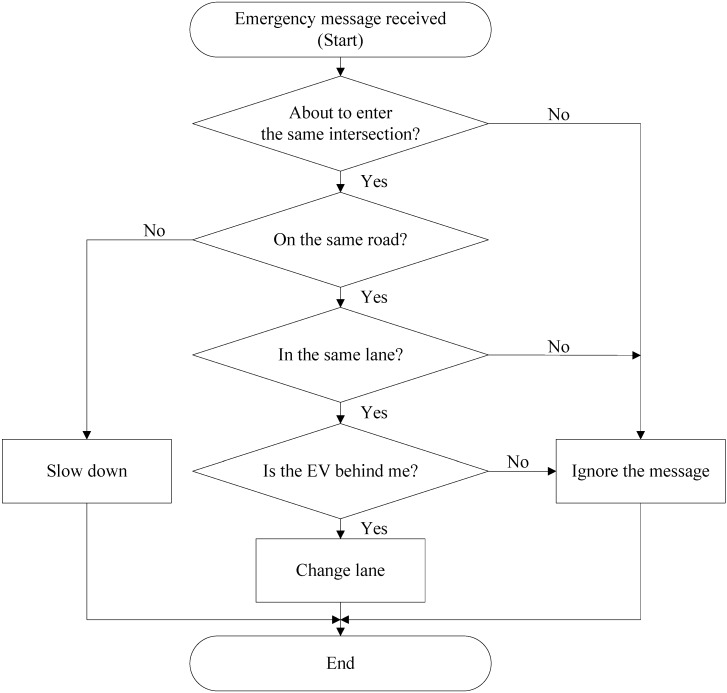
PC process.

In addition to visualizing the position of the EV, other vehicles can ascertain that they may intersect with the EV after receiving emergency messages. The template in Figure 5 shows how vehicles should make reactive decisions after receiving emergency messages.

## 4. Performance Evaluation

### 4.1. Simulation Tools

We used two open-source simulators to test our proposed system: Simulation of Urban Mobility (SUMO, v0.15.0) [33] and the ns-2 network simulator (v2.34) [34]. SUMO is a portable, microscopic simulator that supports map format conversion so that maps such as OpenStreetMap [35] can be imported to simulate realistic road environments. Vehicles in SUMO travel according to Krauss’ car-following model [36], in which speed is increased or decreased based on distances between vehicles; changing lanes and passing vehicles are both permitted. The ns-2 network simulator, which is the most frequently used in wireless *ad hoc* network environments, provides some of the latest communication modules [37]. It also features several wireless signal transmission modules.

**Figure 6:**
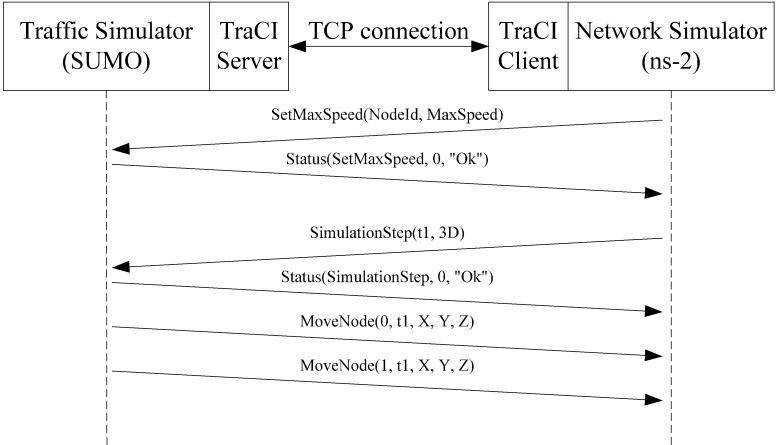
Collaboration between traffic and network simulators.

To support information exchanges within the network simulator, the two simulators must be executed simultaneously and be connected by a traffic control interface (TraCI) [38]. In our tests, the traffic simulator acted as the server and the network simulator as the client, with a TCP connection used for communication between the two (Figure 6). After establishing a TCP connection, the traffic simulator regularly delivers data on the vehicles’ position to update network simulator node locations. The network simulator can demand that the traffic simulator allow specific vehicles to accelerate, decelerate, stop along the side of the road, change lanes, and change destinations; it can also demand the replacement of routine programs for specific traffic lights.

### 4.2. Simulation Environment

**Figure 7:**
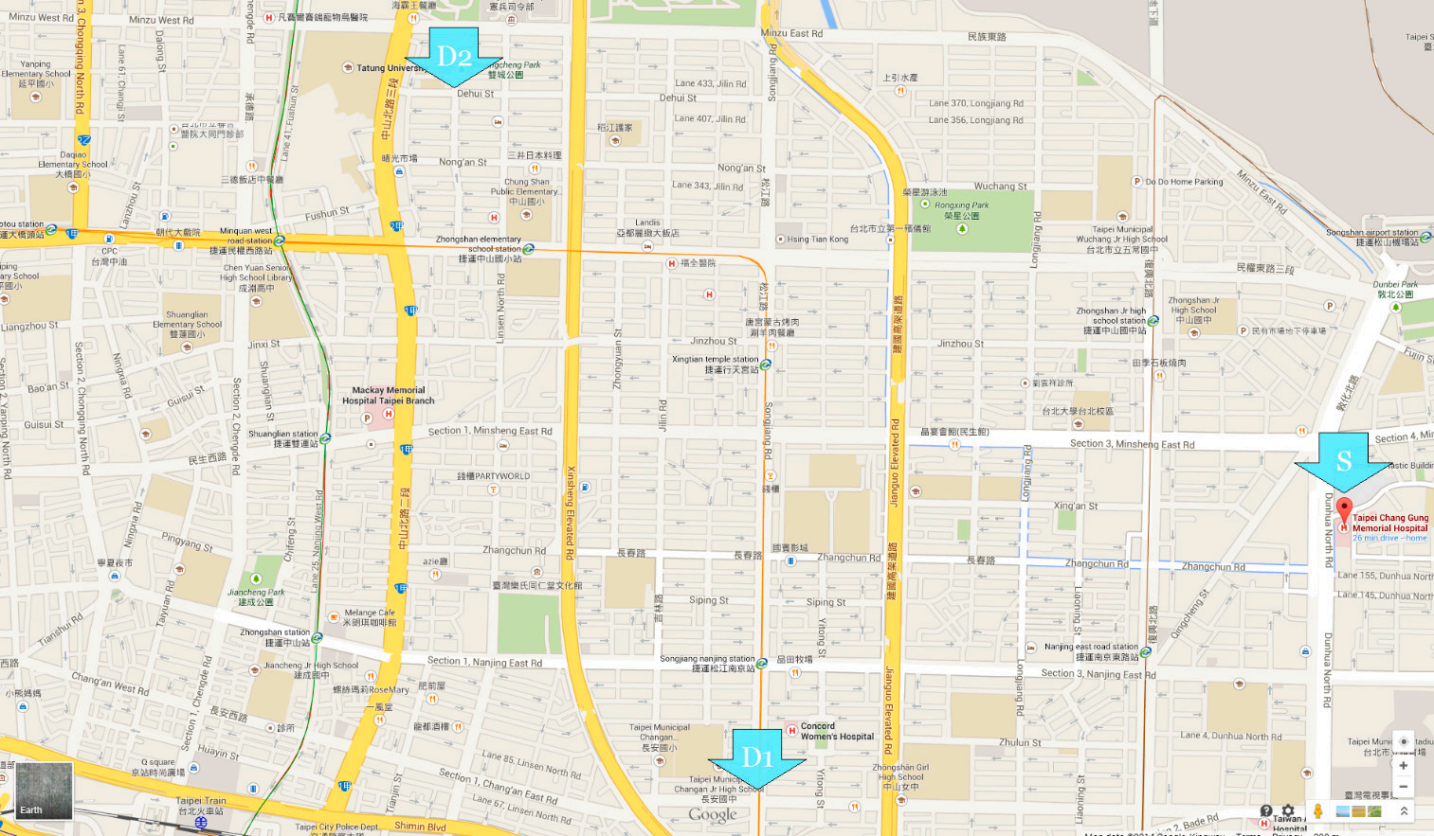
Simulation environment.

To make our simulations more realistic, roads near Chang Gung Memorial Hospital in the Zhongshan District of Taipei were chosen as the real-world environment. The geographic area covered by our map was approximately 2.9 × 2.3 km. Figure 7 shows an image retrieved from Google Maps [39], with the EV starting point indicated by the letter *S*.

Based on the ambulance estimation model developed by Jan de Boer [6], we assumed that the hospital was at the map’s center when establishing a coverage area using isometric graphics. Using Taipei as an example, the area of the city is approximately 272 km^2^. If the 23 hospitals responsible for emergency medical treatment are designated as disaster rescue hospitals, the region of responsibility for each hospital is approximately 12 km^2^ on average (*i.e.*, approximately 2 km radii).

The straight-line distance from the first accident site, *D_1_*, to the EV starting point *S*, was within the radius of responsibility (2 km). The second site, *D_2_*, was slightly outside the radius. The following inequalities were used to estimate the response radii of a hospital.

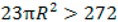
(1)

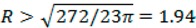
(2)


**Figure 8:**
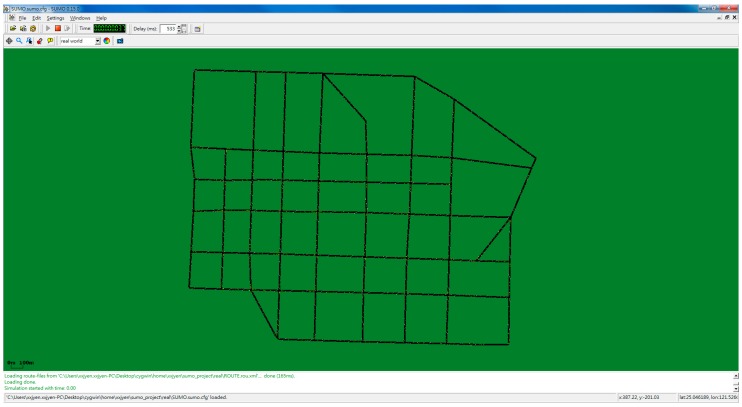
Simulator screenshot (far).

**Figure 9:**
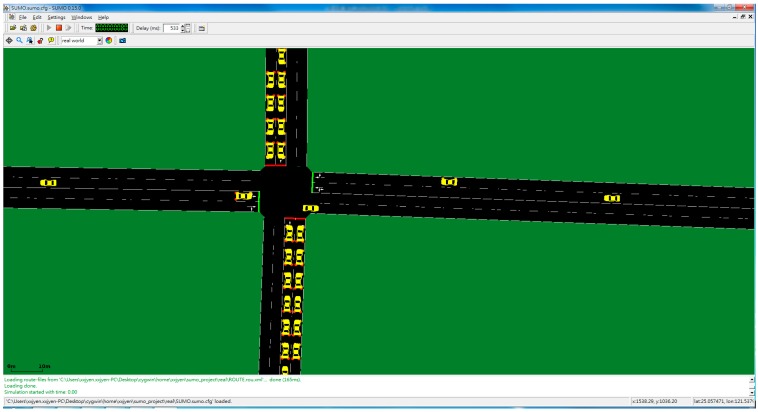
Simulator screenshot (near).

In the following text, the short route denotes the travel path from *S* to *D_1_* and the long route from S to *D_2_*. SUMO screenshots are shown in Figure 8 and Figure 9.

### 4.3. Parameters

Table 1 presents our traffic simulator parameter settings. Vehicle numbers on roads within the map area were 1000, 1500, and 2000. Maximum vehicle speed was approximately 40 km/h for non-EV vehicles and 60 km/h for EVs. The starting point was Chang Gung Memorial Hospital; the two destinations had straight-line distances of 2000 and 2900 meters.

**Table 1:** Traffic simulator parameter settings.

Parameter	Value
Traffic simulator	SUMO 0.15.0
Area of map	2300 × 2900 m^2^
Number of vehicles	1000, 1500 and 2000
Max. speed	EV: 16.67 m/s (60 km/h)
	Vehicles: 11.12 m/s (40 km/h)
TLS building options	default
Length of routes	Short route: 2000 mLong route: 2900 m

**Table 2:** Network simulator parameter settings.

Parameter	Value
Network simulator	ns-2 v2.34
Radio propagation model	Propagation/TwoRayGround
Network interface	Phy/Wireless
MAC protocol	IEEE 802.11 DCF
Max. transmission range	250 m
Warning packet size	256 bytes
Normal packet size	512 bytes
Packets sent by vehicles	1 per second

Parameter settings for the network simulator are shown in Table 2. A two-ray ground reflection model served as the radio propagation model [40]. This model considers both the weakness of straight-line signal propagation and ground signal reflection. The EV broadcast one emergency message per second. All data discussed from this point forward are averages from ten simulations.

### 4.4. Simulation Experiment Design

Function effectiveness was evaluated by three sets of simulations, with a control group of EVs that were not equipped. In order to pass other vehicles to reach specific intersections, EVs must find ways to cross those intersections regardless of traffic light status. Other functions were gradually added to the experimental groups until all three methods were tested. Vehicle and routine traffic light program distributions were fixed within each set, meaning that the only differences among simulations were the EV methods. As shown in the first set of simulations presented in Table 3, the first data item did not use any method; this item served as a baseline. The second data item was the route guidance function (*i.e.*, the path requiring the least amount of time). The third data item was the route guidance and path clearing function, including the dissemination of emergency messages to other vehicles. A comparison of the second and third items revealed the influence of path clearing on the route guidance function. The fourth item consisted of a combination of route guidance and traffic light preemption; a comparison of this and the second item revealed the influence of adding traffic light preemption to the route guidance function. The fifth data item entailed the use of all functions.

**Table 3:** Simulation design.

**Simulation set #1**					
	Item 1	Item 2	Item 3	Item 4	Item 5
RG		V	V	V	V
TLP				V	V
PC			V		V
**Simulation set #2**					
	Item 1	Item 2	Item 3	Item 4	Item 5
RG			V		V
TLP		V	V	V	V
PC				V	V
**Simulation set #3**					
	Item 1	Item 2	Item 3	Item 4	Item 5
RG			V		V
TLP				V	V
PC		V	V	V	V

### 4.5. Simulation Results

#### 4.5.1. Uniform Traffic Settings

**Figure 10:**
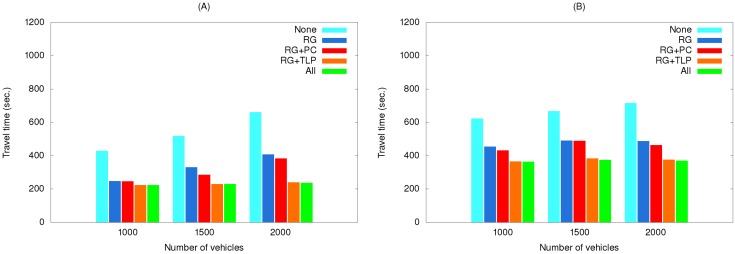
Travel times of simulation set #1 for the (**A**) short route and (**B**) long route.

Figure 10 shows the effects of using various method combinations along the two routes, with route guidance the primary object. When no method was used, the EV encountered congestion at multiple locations. When the route guidance method was used, the EV was able to avoid traffic jams, thereby reducing travel time. Next, the path clearing method included EV communication with other vehicles on the same road, allowing those vehicles to clear a lane for the EV. Time reduction when the path clearing method was included was small for both the short and long routes, since any low-congestion path chosen by the route guidance method diminished the path clearing method’s effectiveness. The reduction in time resulting from traffic light preemption was more obvious because it almost completely eliminated waiting at intersections. Even when the route guidance method found a congestion-free or congestion-reduced path, traffic light preemption still made a significant contribution to time reduction.

**Figure 11:**
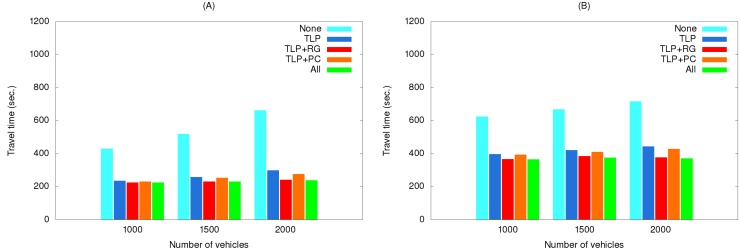
Travel times of simulation set #2 for the (**A**) short route and (**B**) long route.

Figure 11 shows the effects of using various method combinations along the same two routes with traffic light preemption as the primary object. As shown, using this method by itself greatly reduced travel time, but adding other methods to it only contributed minor savings, with route guidance more effective than path clearing.

This is because traffic light preemption reduced the possibilities of the EV and vehicles preceding it being blocked at intersections. As a result, path clearing nearly had no influence on reducing travel time, particularly for low vehicle densities. For high vehicle densities (e.g., 2000 vehicles), the influence is slightly more observable since path clearing allowed vehicles to clear a lane for the EV to quickly pass congested areas. Compared with path clearing, routing guidance could find a path with the shortest travel time, and thus made more contribution to time reduction.

**Figure 12:**
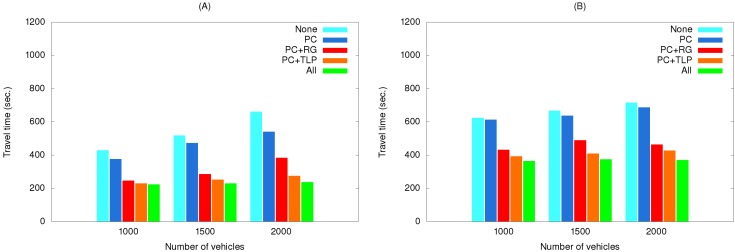
Travel times of simulation set #3 for the (**A**) short route and (**B**) long route.

**Figure 13:**
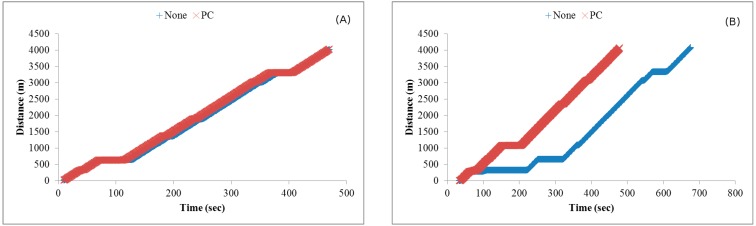
Time-distance relationship obtained from two simulation runs (**A**) and (**B**).

Figure 12 shows the effects of using various method combinations along the two routes with path clearing as the primary object. As shown, the EV did not save much time when this method was used by itself. As a possible explanation, two representative simulation results selected from the third simulation set are shown in Figure 13. The EV time-distance relationship in Figure 13A was observed in most simulation runs due to small differences resulting from vehicles pulling over to the side of the road to allow the EV to pass. However, advantages were erased when the EV encountered a traffic light. In a small number of cases the EV was able to cross the intersection quickly when vehicles yielded in time (Figure 13B).

In summary, the traffic light preemption function was the most effective method, followed by the route guidance and path clearing functions. Travel times increased in all cases where the number of vehicles increased. The system struggled to plan better routes during peak periods and in traffic jams. However, when travel distance increased, the traffic light preemption function performed well because the EV was able to travel at full speed for most of the trip. Using the other methods caused the EV to encounter more disruptions at light-controlled intersections.

#### 4.5.2. Real Traffic Settings

The fixed maximum speeds used in the above simulations are unrealistic assumptions. The reason behind this assumption is that it is not efficient to set the maximum speed of each road segment. In addition, the maximum driving speed of a vehicle in a road segment can vary dramatically in different periods (e.g., rush hours and non-rush hours). To ensure the usefulness of our results, we obtained data from the website of the Taipei Traffic Control Center [41]. The website reports data from a large number of vehicle detectors (VD) deployed throughout the city. Each VD provides real-time traffic information on lane numbers, average numbers of vehicles per time unit, and average vehicle speeds. We collected the information provided by the VDs deployed in the geographic area shown in Figure 7. The average speed data at a rush hour (6 p.m. on 24 September 2014) were used to set maximum speeds for the corresponding road segments.

**Figure 14:**
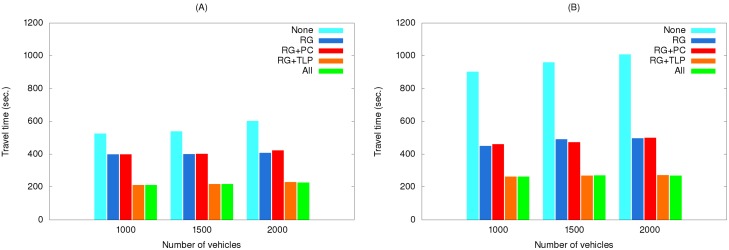
Travel times of simulation set #1 with real traffic data for the (**A**) short route and (**B**) long route.

**Figure 15:**
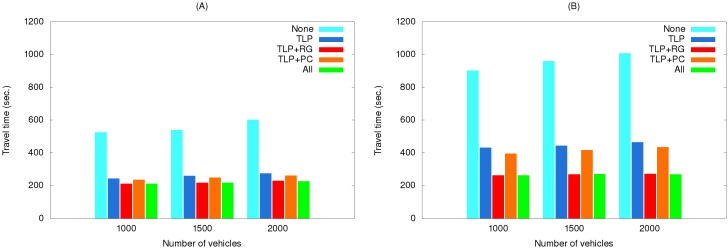
Travel times of simulation set #2 with real traffic data for the (**A**) short route and (**B**) long route.

**Figure 16:**
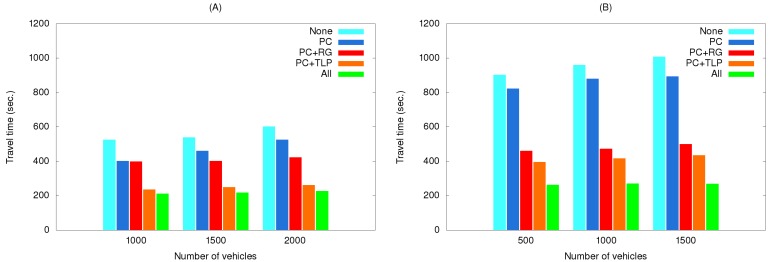
Travel times of simulation set #3 with real traffic data for the (**A**) short route and (**B**) long route.

EV travel times for three simulation sets using real traffic data are shown in Figure 14, Figure 15 and Figure 16. The maximum speeds below 40 km/hour reflect the rush hour scenario, thus explaining the longer travel times compared to those in Figure 10, Figure 11 and Figure 12. However, the same relationships among the various method combinations are similar. Similar relationships were also found for a non-rush hour scenario (1 p.m. on the afternoon of the same date), therefore those data are not presented.

### 4.6. Discussion

#### 4.6.1. Relationship between Travel Time and Cumulative Survival Ratio (CSR)

**Figure 17:**
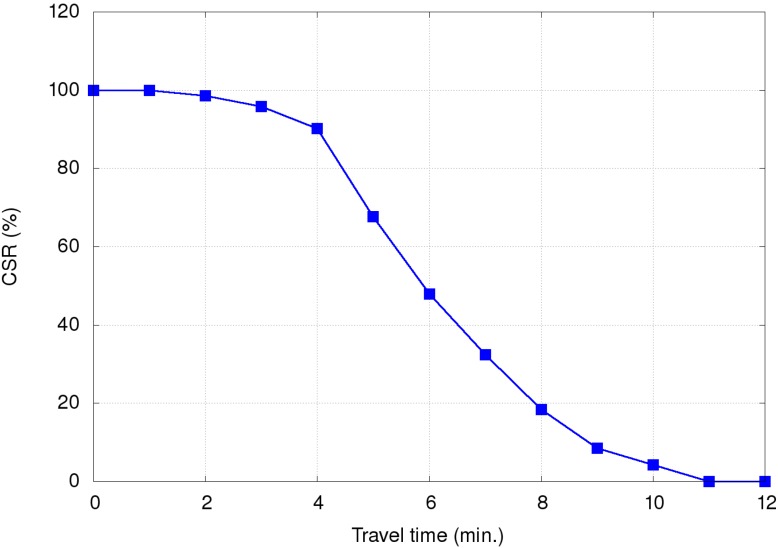
Relationships between travel time and cumulative survival ratio (CSR).

Blackwell and Kaufman [7] collected 5,424 ambulance attendance records for a six-month period from the emergency department of an injury center in a city with a population of 620,000, and calculated a mortality risk of 1.58% for reaction times >5 minutes and 0.51% for reaction times <5 minutes. Table 4 and Figure 17 present data for the relationship between EV travel time and deaths from their study. Cumulative death ratio (CDR*_i_*) is defined as the ratio of the number of deaths when EVs arrive at incident scenes within *i* minutes to the number of total deaths. For example, CDR_3_ can be calculated as 
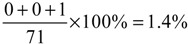
, indicating a CDR of 1.4% if EVs arrive at a specific scene within three minutes. Cumulative survival ratio (CSR*_i_*) is calculated as 1-CDR*_i_*, with results used to estimate improvements from using each method.

**Table 4:** Relationships between travel time, cumulative death ratio (CDR), and cumulative survival ratio (CSR) from Blackwell and Kaufman [7].

Travel Time (s)	Death	CDR (%)	CSR (%)
0~59	0	0	100
60~119	0	0	100
120~179	1	1.4	98.6
180~239	2	4.2	95.8
240~299	4	9.8	90.2
300~359	16	32.3	67.7
360~419	14	52.1	47.9
420~479	11	67.6	32.4
480~539	10	81.6	18.4
540~599	7	91.5	8.5
600~659	3	95.7	4.3
660~719	3	100	0
720+	0	100	0

**Figure 18:**
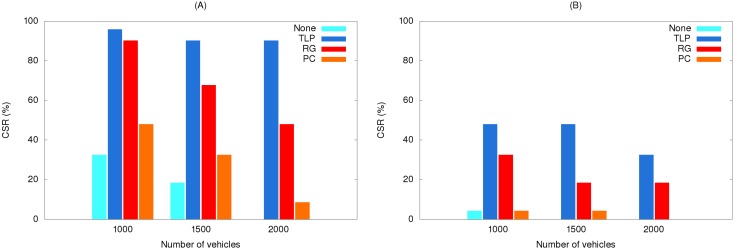
Relationships between various methods and CSR gains for the (**A**) short route and (**B**) long route.

The Table 4 data for EV travel times from our simulations can be used to assess CSRs. Using the results shown in Figure 10, Figure 11 and Figure 12, Figure 18 presents data for relationships between various methods and CSR gains for acute and critically ill patients. Let (*v*_1_, *v*_2_, *v*_3_) denote travel times or CSRs with 1000, 1500 and 2000 vehicles, respectively. When using traffic light preemption with various numbers of vehicles along the short route, the travel time dropped from (427, 517, 661) to (233, 256, 298) s.

As shown in Table 4, the respective CSRs increased from (32.4%, 18.4%, 0%) to (95.8%, 90.2%, 90.2%). For the long route the travel time dropped from (623, 677, 716) to (395, 418, 442) s and CSRs increased from (4.3%, 0%, 0%) to (47.9%, 47.9%, 32.4%). When using route guidance with the same numbers of vehicles along the short route, travel time fell from (427, 517, 661) to (246, 330, 406) s and CSRs increased from (32.4%, 18.4%, 0%) to (90.2%, 67.7%, 47.9%). For the long route, travel time fell from (623, 677, 716) to (453, 490, 486) s and CSRs increased from (4.3%, 0%, 0%) to (47.9%, 18.4%, 18.4%). When using path clearing with the same numbers of vehicles along the short route, travel time was reduced from (427, 517, 661) to (376, 472, 540) s and CSRs increased from (32.4%, 18.4%, 0%) to (47.9%, 32.4%, 8.5%). Long route travel times decreased from (623, 677, 716) to (612, 637, 686) s, and CSRs increased from (4.3%, 0%, 0%) to (4.3%, 4.3%, 0%). From Figure 18, we can observe that no matter which method was used, the CSR improvement is significant for the short route. In contrast, only traffic light preemption shows a great improvement in CSR for the long route. However, we can find that path clearing reduced the EV travel time by 30 s (from 716 to 686 s) for the long route with 2000 vehicles, but the CSR remains 0%. This shows us that reducing the EV travel time does not guarantee increases in CSR.

#### 4.6.2. Difficulty

Path clearing is the hardest method to implement because it requires the installation of a communications device in all vehicles. Presently there are few such vehicles in Taiwan. Further, there are questions about their effectiveness in a country where drivers are not accustomed to yielding to EVs, as they are in western countries. Thus, if such hardware is installed, it must be accompanied by a strong education effort or increased enforcement of existing regulations.

Route guidance is the easiest method to implement, since it can make use of a general road and traffic information resource such as Google Maps with a 3G or 3.5G universal mobile telecommunications system. Information from such a system can be used to calculate proper EV routes. Google has recently introduced a real-time traffic feature, but it uses colors to mark roads with different average speeds separately from its navigation system. Further, there are questions about the accuracy of Google Maps, therefore a mix of that system and data from VDs or closed-circuit cameras may make route guidance more feasible and cost-efficient. Improving the accuracy of real-time traffic information is an ongoing challenge.

Regarding traffic light preemption—the most effective of the three methods described in this paper—the main task is to manage remote control interfaces with ITS servers and EVs. One potential problem is the fairly rare scenario of two or more EVs entering the same intersection at the same time. A solution would entail prioritizing emergency messages.

## 5. Conclusions

Numerous researchers have attempted to use VNs to shorten EV arrival times, but the majority of studies have focused on communications technologies and protocols rather than assessing actual VN effectiveness in EMS management. We combined several existing VN applications. The implementation system used in this study had three functions: route guidance, traffic light preemption, and path clearing. Our simulation results indicate that traffic light preemption is the best method for significantly increasing CSRs for acute and critically ill patients (from 32.4% to 90.2%). The second most effective method was route guidance (14.1% and 57.8%), followed by path clearing (0% and 15.5%). Our data also confirm increased travel times and reduced CSRs during peak traffic periods and traffic jams, therefore municipalities must continue to determine the best and most efficient distributions of medical resources.

Due to space limitations, we narrowed our focus to medical outputs for different approaches designed for emergency services with the assistance of VNs. The effects of radio propagation models, car moving patterns, street topologies, and other parameters were not addressed in this study. Our plans include adding a radio propagation model [42,43,44] to our simulations.
